# Amino acids regulating skeletal muscle metabolism: mechanisms of action, physical training dosage recommendations and adverse effects

**DOI:** 10.1186/s12986-024-00820-0

**Published:** 2024-07-02

**Authors:** Guangqi Li, Zhaojun Li, Junyi Liu

**Affiliations:** 1 School of Physical Education, Northeast Normal university, No. 5268, Renmin Street, Changchun city, Jilin province 130024 People’s Republic of China; 2 Gaomi Municipal Center for Disease Control and Prevention, Gaomi city, Shandong People’s Republic of China

**Keywords:** Muscle protein synthesis, Amino acid supplementation, Branched-chain amino acids, Creatine, Glutamine, β-alanine

## Abstract

Maintaining skeletal muscle mass is important for improving muscle strength and function. Hence, maximizing lean body mass (LBM) is the primary goal for both elite athletes and fitness enthusiasts. The use of amino acids as dietary supplements is widespread among athletes and physically active individuals. Extensive literature analysis reveals that branched-chain amino acids (BCAA), creatine, glutamine and β-alanine may be beneficial in regulating skeletal muscle metabolism, enhancing LBM and mitigating exercise-induced muscle damage. This review details the mechanisms of these amino acids, offering insights into their efficacy as supplements. Recommended dosage and potential side effects are then outlined to aid athletes in making informed choices and safeguard their health. Lastly, limitations within the current literature are addressed, highlighting opportunities for future research.

## Introduction

Amino acids are the building blocks for all life forms. There are 21 amino acids that make up human tissue proteins, with selenocysteine now included in the list and utilized for synthesizing proteins and essential molecules that fulfill significant physiological functions related to nutrition, sensory perception, and biological regulation [1–3]. These amino acids can be classified into essential amino acids (EAA, incapable of endogenous synthesis and must be obtained through dietary intake) and non-essential amino acids (NEAA, synthesized within the cell) [4]. Furthermore, under specific circumstances such as physical activity, lactation, trauma, and illness, certain NEAA may experience heightened utilization rates, thereby being referred to as conditionally essential amino acids (CEAA), when their demand exceeds the synthesis capacity [5]. Thus, amino acids, particularly EAA or CEAA, are commonly consumed as nutritional supplements by athletes across all levels of sports participation and individuals engaged in regular and moderate physical activity.

Participation in physical activity is a cornerstone of a healthy lifestyle and represents an excellent, cost-effective means to enhance overall well-being. However, engaging in physical activity can result in immediate decreases in muscle strength and the development of exercise-induced muscle damage, characterized by symptoms such as muscle swelling, stiffness, and discomfort [6, 7]. Skeletal muscles, which constitute approximately 45–55% of total body weight, are crucial for performance, locomotion [8]. Muscle metabolism is contingent upon the intricate equilibrium between muscle protein synthesis (MPS) and muscle protein breakdown (MPB). A surplus of MPS over MPB leads to a net positive protein balance, facilitating muscle growth, whereas the opposite scenario leads to muscle atrophy [9]. Maintaining a favorable net muscle protein balance, achieved by elevating MPS or reducing MPB, is paramount to improve function and strength. For example, dietary constraints, such as energy restriction, can curtail the rate of MPS, leading to a negative net protein balance and consequent lean body mass (LBM) loss, underscoring the potential importance of protein or amino acid supplementation to counteract negative net protein balance during dietary energy limitations [10–12].

A comprehensive analysis of the available literature reveals that supplementation with BCAA, creatine, glutamine, and β-Alanine has shown promise in increasing LBM, enhancing muscle strength and functionality. Although creatine, glutamine, and β-Alanine may modulate MPS and MPB indirectly, their additional effects are vital for sustaining muscle metabolism and mitigating muscle damage and fatigue. For example, creatine acts as a cellular energy buffer, maintains mitochondrial integrity, increases phosphocreatine (PCr) availability and mitigates oxidative damage [13]; glutamine acts as a nitrogen transporter, is a precursor of nucleotide bases and the antioxidant glutathione, and it regulates acid-base balance [14]; β-Alanine is a precursor for carnosine (β-alanyl-L-histidine) synthesis, which is involved in pH-buffering, calcium regulation, and endogenous antioxidant activity [15]. Hence, this review primarily focuses on elucidating the mechanisms of action, recommended dosages and potential side effects associated with these amino acid supplements in disease-free populations. The following sections offer valuable insights into amino acid supplementation for athletes and those engaged in regular moderate-vigorous physical activity to enable informed decision-making. Furthermore, this review addresses the existing gaps in the literature, emphasizing the imperative need for further research.

### Branched-chain amino acids (BCAA)

BCAA refers to a class of amino acids characterized by their aliphatic side chains, including three specific amino acids: leucine, isoleucine and valine [16]. Structurally, the BCAA exhibit a high degree of hydrophobicity compared to other proteogenic amino acids, a property critical for preserving the maturity and stability of folded proteins [17]. The three amino acids found in the BCAA group are categorized as EAA, indicating that they cannot be endogenously synthesized in the human body and must be obtained through dietary sources to support vital physiological functions [4]. In contrast to other amino acids primarily metabolized in the liver, BCAA are absorbed and catabolized within skeletal muscle [18, 19]. This distinctive characteristic is crucial for the pivotal roles of BCAA in MPS and post-exercise muscle recovery [19]. Given the recognition of BCAA as a key regulator in MPS, these amino acids have become widely popular as nutritional supplements for individuals engaged in physical activity.

### The potential mechanism of BCAA in regulating skeletal muscle metabolism

BCAA are integral to the regulation of skeletal muscle protein metabolism, through a complex network of intracellular signaling pathways. The mammalian target of rapamycin (mTOR) signaling pathway is a key driver of protein synthesis with eukaryotic translation initiation factor 4E (eIF4E) binding protein 1 (4E-BP1) and S6 kinase 1 (S6K1) playing key roles as phosphorylated substrates of mTOR complex 1 (mTORC1) [20–25]. These proteins regulate mRNA translation initiation and elongation, ultimately facilitating protein synthesis [24, 26]. Stress response proteins called Sestrins, including Sestrin1, Sestrin2 and Sestrin3, function as upstream negative regulators of mTORC1. The Sestrins have been shown to interact with GTPase-activating protein toward rags 2 (GATOR2), leading to the inhibition of mTORC1 activity [27]. The interaction between intracellular Sestrins and GATOR2 depends on amino acid availability. In vivo studies have demonstrated specific binding sites for leucine within Sestrin1 and Sestrin2, but not Sestrin3, and ingestion of leucine can thereby activate mTORC1 through these signaling pathways [28–30]. Further investigation is required to elucidate the precise mechanism by which leucine binding to Sestrin1/2 disrupts their interaction with GATOR2.

In addition to the role of Sestrins, research indicates that leucyl tRNA synthetase (LRS) functions as a sensor for leucine, activating the mTORC1 pathway. Specifically, LRS serves as a GTPase-activating protein (GAP) for RagD GTPase, prompting its transition from the inactive GTP-bound form (RagDGTP) to the active GDP-bound state (RagDGDP). Rag GTPases are known to be pivotal mediators in the amino acid-responsive mTORC1 pathway. Leucine induces the translocation of mTORC1 to the lysosome, where GTP-bound RagB-containing Rag heterodimers interact with mTORC1, leading to the activation of mTORC1 [31–33].

β-hydroxy-β-methylbutyrate (HMB), a metabolite derived from leucine, represents approximately 5–10% of leucine metabolism within the body [34]. Numerous studies support that HMB can stimulate MPS while simultaneously reducing MPB [35–39]. Mechanistically, HMB activates the mTORC1 pathway by enhancing AKT phosphorylation, which subsequently inactivates the tuberous sclerosis complex 2 (TSC2) [40, 41]. The TSC1-TSC2 complex acts as a negative regulator of mTORC1, with TSC2 serving as a GTPase-activating protein (GAP) for the small Ras-related GTPase Rheb, while TSC1 aids in stabilizing and safeguarding TSC2 from degradation [24]. The activated GTP-bound form of Rheb directly interacts with mTORC1 or enhances substrate recognition by mTORC1, thereby activating the mTORC1 pathway [23]. Additionally, increased AKT phosphorylation induces the phosphorylation of Forkhead box O 1 (FOXO1) and decreases nuclear FOXO1 levels, ultimately leading to the downregulation of muscle atrophy-related muscle RING-finger protein-1 (MURF1), thus mitigating MPB [41, 42].

**Fig. 1:**
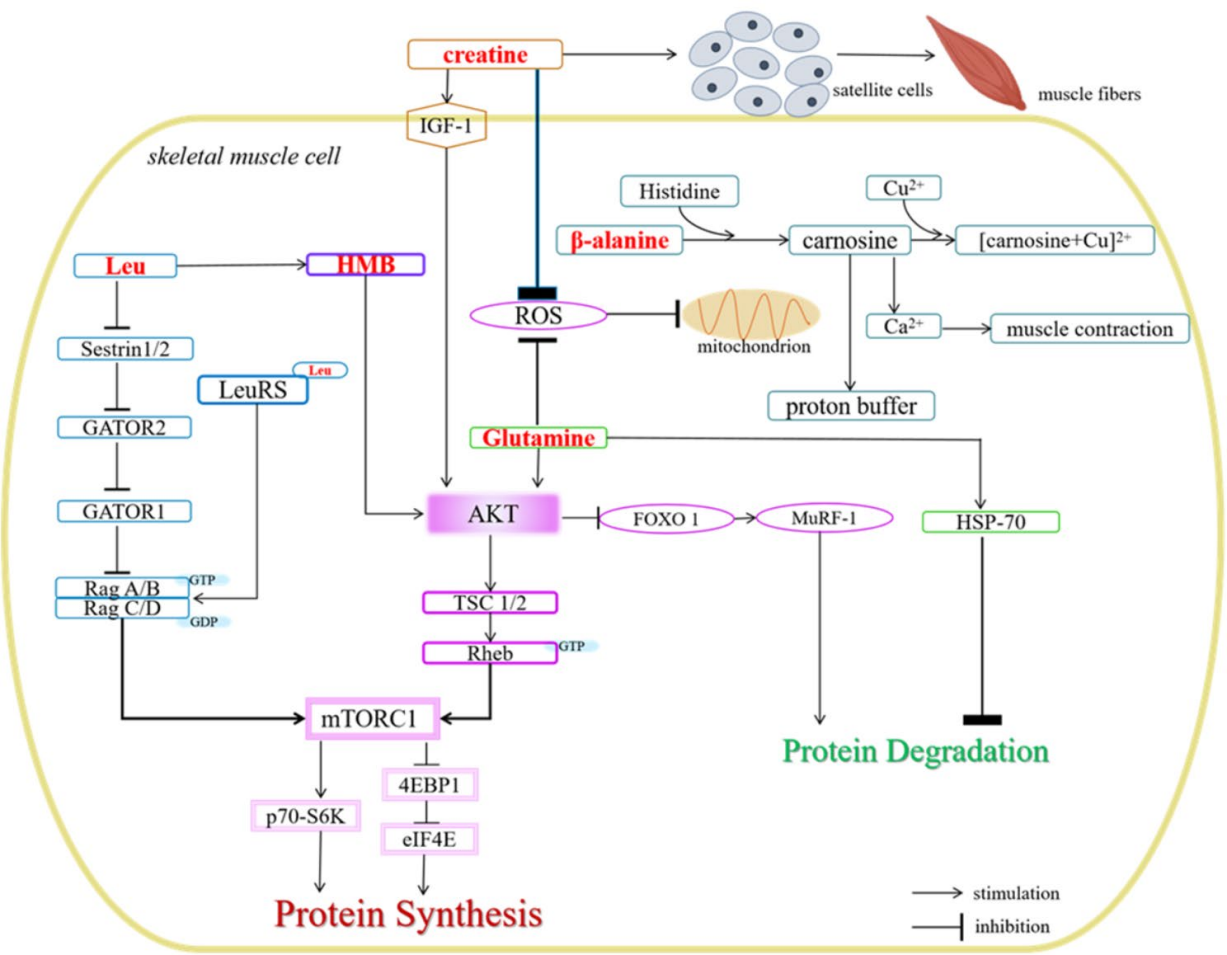
The mechanisms of amino acids involvement in skeletal muscle cell metabolism. Leucine activates the mTORC1 pathway through the Sestrin1/2-GATOR2, LRS, or the dephosphorylation of AKT by its metabolite, HMB. Creatine upregulates IGF-1 and MRFs expression, activates satellite cells, and mitigates mitochondrial damage. Glutamine may activate AKT signaling pathways, mitigate mitochondrial damage and activate HSP-70. β-alanine is is a rate-limiting reactant in the synthesis of carnosine. Carnosine act as an intracellular pH buffer, metal-ion chelator, and regulator of calcium release and calcium sensitivity

In summary, leucine activates the mTORC1 pathway through various mechanisms (Fig. 1), including the disruption of the Sestrin1/2-GATOR2 complex, the activation of LRS and the dephosphorylation of AKT facilitated by its metabolite, HMB.

### Dosage recommendations and adverse effects

Utilizing the indicator amino acid oxidation (IAAO) method, the recommended total intake of BCAA for adults is 0.144 g/kg/day, with specific leucine, isoleucine, and valine intake at 0.055 g/kg/day, 0.042 g/kg/day, and 0.047 g/kg/day, respectively [43]. Furthermore, the recent study conducted by the European Prospective Investigation into Cancer and Nutrition (EPIC) indicates that, on average, adults consume 5.6 g of leucine, 3.2 g of isoleucine and 3.8 g of valine daily, with leucine being the most consumed EAA [44].

Leucine is recognized as a most potent and widely used amino acid supplement, due to its central role as the primary driver of MPS. Multiple studies have suggested that supplementation with low doses of protein or EAA enriched with leucine result in greater MPS stimulation compared to the consumption of 20–40 g of whey protein [45–48]. For example, the ingestion of just 3 g of EAA enriched with 1.2 g of leucine can elicit MPS to a level equivalent to that achieved by consuming 20 g of whey protein in older women, both at rest and following exercise [47]. In addition, D J Wilkinson et al. have reported that supplementation with 3 g of leucine in isolation can induce maximal MPS stimulation [46].

There is a debate that the consumption of leucine in isolation may be more efficacious for MPS than BCAA supplementation due to leucine’s competition with other neutral amino acids for transport into muscle cells, potentially limiting its unique impact on MPS [49]. However, supplementation with leucine alone can result in decreased concentrations of isoleucine and valine in both plasma and muscle [50–52]. This occurrence is attributed to ketoisocaproate, a product of leucine transamination, which enhances BCKA dehydrogenase activity in muscles, liver, and adipose tissues [50–53]. Therefore, the potential limitation of MPS induced by leucine intake alone due to the bioavailability of isoleucine and valine underscores the necessity for rigorously controlled double-blind trials to thoroughly assess and contrast the impact of leucine in isolation versus BCAA supplementation on MPS in individuals, both in sedentary conditions and during physical activity.

Although HMB can stimulate MPS and reduce MPB, several systematic reviews and meta-analyses have shown that HMB supplementation does not increase or prevent loss of muscle mass [35, 54–56]. Furthermore, HMB supplementation did not enhance the increases in muscle mass and strength resulting from resistance training [35].

The analysis of data from the International Council on Amino Acid Science has resulted in the determination of a tolerable upper intake level for leucine. The recommended limit for leucine supplementation is set at 0.5 g/kg/day for adults and 0.43 g/kg/day for elderly men over 70 years of age, corresponding to daily intakes of 35 g and 30 g, respectively, for a 70 kg individual [2, 57, 58]. Exceeding these established values can lead to elevated blood ammonia levels primarily as a result of the rapid transamination rates [2]. This heightened transamination activity is attributed to the higherKm values (Michaelis-Menten constant) of BCAA aminotransferases compared to tissue BCAA, being two to four times greater [19].

### Limitations and future research opportunities

Based on existing research, the efficacy of leucine supplementation in maintaining protein balance over extended periods remains inconclusive [59–61]. Consequently, future research is necessary to evaluate the impact of prolonged BCAA or leucine supplementation on muscle anabolism, taking into account factors such as age, type of training, and duration.

BCAA are commonly included in a variety of supplements, including whey protein. However, current evidence does not support BCAA or leucine supplementation for muscle hypertrophy in individuals who meet daily protein intake recommendations (≥ 1.6 g/kg/day) for exercise, suggest that supplementation with BCAA may increase muscle mass in individuals who cannot guarantee sufficient protein intake [62–65]. Moberg et al. reported that the hierarchy of anabolic stimulation for whole intact proteins is as follows: EAA > BCAA > leucine alone, with the condition that sufficient leucine is present [66]. As such, the potential benefits of BCAA supplementation for athletes, including enhanced muscle function, reduced muscle soreness and delayed fatigue, should be approached with caution and warrant further investigations.

### Creatine

Creatine is one of the most widely used nutritional supplements among both elite and amateur athletes [67, 68]. Under typical circumstances, an average individual requires approximately 2 g of creatine per day to offset daily losses, which equates to roughly 1.7% of the total creatine content in the body [69]. Creatine is sourced from two origins: one is endogenous synthesis (about 1 g, approximately half of the daily requirement) in the pancreas, liver and kidneys from methionine, glycine and arginine metabolism. The other source is exogenous from food like beef and seafood [70]. The average total creatine storage capacity in the human body is about 120 g, with the majority (95%) located in skeletal muscles and the remaining 5% distributed among the brain, kidneys, liver, and testes [71]. In skeletal muscle tissue, creatine is found in two primary forms: approximately 33% exists as the unbound free form, while the remaining 67% binds to phosphate molecules released from adenosine triphosphate (ATP)to form PCr. PCr, with the assistance of creatine kinase (CK), facilitates the transfer of phosphate to adenosine diphosphate (ADP) to regenerate ATP [71, 72]. Consequently, creatine operates as an energy regulator by promoting the recycling of ATP, exerting significant influence on muscle recovery, improving LBM, and enhancing strength [73].

As is well known, creatine supplementation enhances resistance training adaptations [68, 74, 75]. Studies have shown that skeletal muscle contraction stimulates intramyocellular uptake of creatine, and that a combination of creatine supplementation and resistance training may maximize its accretion [76]. As results, creatine supplementation combined with resistance training increases LBM and muscle strength [75, 77–80]. However, there is no evidence to suggest that creatine supplementation independent of resistance training can increase muscle strength.

### The potential mechanism of creatine in regulating skeletal muscle metabolism

Creatine may regulate skeletal muscle metabolism through several pathways.Firstly, it may accelerate MPS by upregulating insulin-like growth factor-1 (IGF-1) levels and enhancing the release of IGF-1 from muscle cells [81, 82]. IGF-1 functions as a ligand, binding to IGF receptors on the cell membrane and subsequently activating signaling pathways, including the AKT/mTOR pathways, which promote cell growth and proliferation [83]. For instance, in a double-blind cross-over study involving high-intensity resistance exercise following five days of creatine supplementation, researchers observed a 30% increase in IGF mRNA expression in resting muscle, which leads to elevated phosphorylation levels of 4E-BP1, and enhancing MPS [81]. Secondly, creatine supplementation upregulates mRNA and protein expression of myogenic regulatory factors (MRFs), such as myogenin and MRF-4 [84]. These MRFs play pivotal roles in the activation, proliferation, and differentiation of satellite cells (skeletal muscle resident stem cells), which are crucial for the repair and regeneration of muscle fibers [71]. Satellite cells, located between the sarcolemma and the basal lamina of muscle fibers, fuse with the muscle fiber membrane upon activation, passing through the sarcolemma and differentiating into myonuclei, ultimately boosting muscle fiber protein synthesis [85]. In a study, the administration of creatine subsequent to resistance training was found to increase satellite cell numbers and myonuclei concentration in human skeletal muscle fibers, thereby facilitating muscle fiber hypertrophy [86].

Several studies indicate that creatine supplementation diminishes urinary 3-methylhistidine (3-MH, a global marker of protein breakdown) and leucine oxidation (a marker of MPB), implying a potential reduction of protein catabolism [87, 88] Mechanistically, the regulation of MPB by creatine may be attributed to its ability to attenuate mitochondrial damage. It is well-established that the aging process and physical activity can negatively impact mitochondrial function, resulting in compromised respiratory chain activity and generated reactive oxygen species (ROS) [89, 90]. ROS can accelerate muscle fatigue and damage by impairing calcium sensitivity and altering cell membrane permeability [90, 91]. Creatine reduces ROS, which mitigates mitochondrial damage, thereby protect against muscle damage and MPB [92]. The effects may arise from creatine’s direct antioxidant properties or its role in enhancing cell membrane stability and cellular energy production [13].

Taken together, existing research suggests potential mechanisms by which creatine may augment MPS and diminish MPB (Fig. 1), such as the upregulation of IGF-1 and MRFs expression, activation of satellite cells, and mitigation of mitochondrial damage. However, the precise mechanism by which creatine regulates skeletal muscle metabolism remains to be further determined.

### Dosage recommendations and adverse effects

As previously mentioned, research has shown that creatine supplementation can improve exercise performance, muscle strength and muscle mass, especially in aging individuals [74, 75]. Due to the inadequate endogenous synthesis of creatine in the body, exogenous supplementation is often necessary to optimize anabolism in the majority of individuals [70]. In order to sustain proper levels of creatine in muscle tissue, it is advised to consume 1–3 g of creatine daily through dietary sources, a recommendation that especially important for vegetarians, who typically have lower muscle creatine stores and may experience benefits from creatine supplementation [93, 94]. Wallimann and colleagues recommend the inclusion of 3 g/day of exogenous creatine in individuals’ diets to promote overall health, particularly with advancing age [95]. Due to the benefits creatine provides, such as enhancing acute exercise capacity and training adaptations, elite athletes are frequently advised to utilize creatine supplementation as an ergogenic aid to optimize their training results [68].

After a saturation period, creatine supplementation can enhance high-intensity performance and strength/power by 5–15%, correlating with the degree of increase in muscle PCr levels [96]. The most rapid and effective approach to achieve creatine saturation entails the consumption of 20 g/day (or 0.3 g/kg/day) of creatine, administered in four 5 g doses, over a period of 5–7 days. Subsequently, a dose of 3–5 g/day (or 0.03 g kg/day) is advised for the maintenance of elevated muscle creatine stores [76, 97]. Studies indicate that a daily intake of 5–10 g of creatine is necessary for larger athletes to sustain increased creatine [96, 98, 99]. It is generally observed that heightened muscle creatine levels will return to baseline within 4–6 weeks after supplementation cessation [99, 100]. While, there is a lack of empirical evidence, it is unlikely that muscle creatine levels would fall below baseline levels post-supplementation discontinuation, indicating that prolonged exogenous creatine use does not inhibit the body’s inherent creatine synthesis capacity [101, 102].

Since the early 1990s, creatine monohydrate extensively researched and continues to be the predominant form of creatine supplementation [67]. Various newer forms of creatine, such as creatine dipeptides, creatine salts, and creatine complexes with other nutrients, have emerged in the market, claiming superior efficacy, safety, and bioavailability compared to creatine monohydrate [103]. Nevertheless, none of these alternative forms of creatine demonstrate the exceptional absorption rate of creatine monohydrate, which is estimated to be around 99% [104]. Various forms of creatine have a lower content per gram in comparison to creatine monohydrate, which boasts a creatine content of 87.9%, suggesting that creatine monohydrate may offer superior bioavailability [105]. Despite its limited solubility, creatine monohydrate powder demonstrates stability and resistance to degradation into creatinine even under prolonged storage and elevated temperatures [105]. Consequently, creatine monohydrate powder continues to be the favored option, prompting the need for additional research to formulate a stable and efficacious liquid form of creatine supplementation.

The prevalence of creatine use in sports and exercise, has prompted inquiries into potential side effects linked to its supplementation. Available studies have indicated that creatine supplementation, at doses ranging from 0.3 to 0.8 g/kg/day for up to 5 years, poses no adverse health risks, regardless age [68]. While anecdotal reports have suggested potential side effects like water retention, muscle cramping, dehydration, and kidney dysfunction. However, multiple studies have shown that creatine supplementation is not associated with increased instances of muscle cramping or dehydration [106–111]. Publications by the International Society of Sports Nutrition in 2007 and 2017 consistently confirm the efficacy and safety of creatine monohydrate [68, 104, 112, 113].

In summary, when administered at recommended dosages, creatine supplementation is deemed safe and advantageous for individuals spanning a wide range of demographics, including both healthy individuals and those with medical conditions across various age brackets from infancy to old age.

### Limitations and future research opportunities

Several studies have indicated that the concurrent consumption of creatine with carbohydrates or a combination of carbohydrates and protein can improve creatine absorption [114, 115]. However, when compared to the use of creatine in isolation, this combined approach only marginally elevates muscle creatine levels and does not impact exercise performance or training adaptations significantly [68]. The potential effects of this combined method on muscle mass are still inconclusive, and existing evidence does not support its recommendation as a viable strategy.

In comparison to resistance exercise, creatine supplementation may not exert a substantial influence on continuous, prolonged, steady-state moderate intensity endurance exercise, but may be beneficial in higher intensity activities [70, 116, 117]. Further research is warranted to explore the effects of creatine supplementation on diverse endurance modalities.

Several reports have demonstrated enhancements in muscle mass, strength and performance in various female populations with creatine supplementation, including young, elderly, and post-menopausal women [118, 119]. However, females may be less responsive to exogenously creatine supplementation due to the presence of the higher baseline intramuscular concentrations in their muscles [120–122]. The optimal dosage of creatine supplementation for females compared to males remains uncertain.

### Glutamine

Glutamine is a non-essential amino acid predominantly biosynthesized in skeletal muscles, with a smaller fraction originating from organs such as the lungs, brain, liver and adipose tissues [123, 124]. During the biosynthesis of glutamine within skeletal muscle, BCAA undergo deamination, transferring amino groups to α-ketoglutaric acid to produce glutamic acid. Subsequently, glutamic acid combines with ammonia to form glutamine through the catalytic activity of glutamine synthetase [123]. Glutamine is one of the most abundant amino acids found in human tissues and organs, comprising 80% of free amino acids in skeletal muscles and 20% in plasma [125].

Functionally, glutamine plays a vital role as a nitrogen transporter, aiding in the transfer of nitrogen generated in tissues to the kidneys for excretion, including the nitrogen released by skeletal muscles, with more than 80% of it being transported through glutamine [126, 127]. Glutamine serves as a critical source of ammonia necessary for the kidneys to regulate acid-base balance, while also serving as an energy source for various cell types such as epithelial cells, fibroblasts, and immune cells [128, 129]. Additionally, it is a precursor for the biosynthesis of proteins, amino acids, nucleotides, and glucose [130]. Research indicates that approximately 30–35% of the nitrogen resulting from protein degradation exists in the form of glutamine, underscoring its fundamental role in protein structure [131].

Notably, levels of glutamine in both blood and skeletal muscle decline rapidly during different forms of physical activity (e.g., resistance training and exhaustive exercise) [132–134]. These findings underscore that the potential inadequacy of endogenous glutamine synthesis in meeting the needs of athletes and individuals with specific medical conditions. Consequently, glutamine is recognized as a CEAA, that plays a role in immune system function, intestinal health, and overall protein balance [135, 136].

Several research has been reported that glutamine supplementation can improve MPS, decrease MPB, expedite wound healing, and mitigate muscle damage in animal models [137–146]. For example, increasing glutamine from 0.67 to 5.0 mM led to a 66% increase in MPS in rat skeletal muscle, even without insulin. In the presence of insulin, protein synthesis was boosted by 80% [137]. Their findings also revealed that the addition of 15 mM glutamine significantly reduced net protein loss and MPB compared to levels observed in the absence of glutamine [147]. Additionally, glutamine supplementation has been shown to stimulate protein synthesis and inhibit protein degradation in the mucosal cells of the small intestine [148, 149]. However, there remains a contentious discourse surrounding the impact of glutamine on MPS and MPB in human subjects. While some studies indicate that glutamine supplementation does not significant influence LBM and body composition, others have found a positive association between muscle glutamine levels and MPS in humans, implying a potential role for glutamine in muscle protein metabolism [150–154]. Further investigation is needed to elucidate the potential role of glutamine in regulating muscle protein turnover in humans.

### The potential mechanism of glutamine in regulating skeletal muscle metabolism

According to existing research, glutamine may enhances net muscle mass through several mechanisms: (I) Glutamine may activates the AKT/mTOR signaling pathway through upregulation levels of AKT, mTOR and 4E-BP1 and downregulation ubiquitin ligases atrogin-1 (MAFbx) and MuRF-1 [155, 156]. (II) Glutamine directly stimulates glycogen synthesis through the activation of glycogen synthase, thereby facilitating energy production for MPS [154, 157, 158]. (III) Glutamine functions as the main nitrogen transporter in the body, thereby mitigating the buildup of harmful ammonia and its byproducts within muscle tissues [159, 160]. The presence of ammonia and its metabolites has been shown to interfere with enzyme activity, ion permeability, and the NAD+/NADH ratio in cells [161]. (IV) Glutamine stimulates the upregulation of heat shock proteins (HSP), specifically HSP70, via the processes of O-glycosylation and phosphorylation of transcription factors heat-shock factor 1 (HSF-1) and specificity protein 1 (Sp1) [162, 163]. HSP70 acts as a molecular chaperone, regulating protein folding, ubiquitin degradation pathways, and subcellular protein localization, thereby safeguarding against cell damage [164, 165].

Glutamine is recognized as an indirect antioxidant due to its role in glutathione synthesis, which aids in the elimination of ROS and shields against muscle damage [145]. Thus, it may regulate muscle plasticity, prevent muscle damage and promote muscle recovery [146, 166, 167].

Collectively, glutamine may increase MPS and reduce MPB through various potential mechanisms (Fig. 1), including the activation of AKT signaling pathways, stimulation of glycogen synthesis, up-regulation of the HSP family expression, glutathione synthesis, and reduction of ammonia and its metabolite accumulation. However, most studies on glutamine are based on animal models, and translating these findings to human trials may not be straightforward. Further investigation is needed to better understand the precise mechanisms of glutamine in skeletal muscle in humans.

### Dosage recommendations and adverse effects

Given the current uncertain evidence of glutamine in muscle protein metabolism, body composition, and athlete performance, well-controlled clinical trials are needed to elucidate definitive guidelines for the supplementation of glutamine. The recommended daily intake of glutamine from dietary protein for a 70 kg individual is 3–6 g, while supplementation doses during exercise can range from 2 g to 40 g per day (0.05–0.6/kg/day) [14, 49, 125]. A review by Novak et al. showed that 0.2 g/kg/day of glutamine is the minimum concentration required for detectable clinical results [168]. It is worth noting that low plasma glutamine levels have been positively correlated with certain types of exercise, particularly those that are prolonged or exhaustive activities, indicating potential benefits of glutamine supplementation for individuals engaging in very strenuous exercise [132–134]. For instance, glutamine supplementation (10 g/day for 30 days) improved knee muscle power, strength, glycemic regulation, and plasma redox balance in elderly women aged 60–80 who were physically active [169].

It is essential to note that around 70% of orally administered glutamine undergoes degradation in the small intestine, resulting in only approximately 30% entering the bloodstream [5, 170]. To improve the bioavailability of glutamine and mitigate its instability, L-alanyl-L-glutamine, a dipeptide comprising glutamine and alanine, has been employed in lieu of free glutamine among athletes. Further research indicates that L-alanyl-L-glutamine (0.2 g/kg/day) exhibits superior stability and efficacy in elevating plasma and muscle glutamine levels, thereby promoting MPS in comparison to free glutamine [171, 172].

Glutamine is widely regarded as safe and well-tolerated for healthy individuals at recommended doses [14]. Research indicates that even higher glutamine supplementation levels, as high as 50–60 g/day or 0.65 g/kg/day for the short-term were safe and did not result in adverse events in hospital patients [173, 174]. Therefore, the safe upper intake level of glutamate in healthy individuals still needs further investigation.

### Limitations and future research opportunities

Given the decline in plasma glutamine levels during prolonged or intense physical exertion, glutamine supplementation may offer advantages for individuals engaging in strenuous exercise. In addition, glutamine has a unique property of rapid turnover rate, indicating that its role in cellular metabolism cannot be underestimated. Nonetheless, the instability of free glutamine and its substantial degradation by the small intestine present obstacles, making the dipeptide form, L-alanyl-L-glutamine, may be as a more feasible option in commercial supplementation products. Therefore, future research, particularly in the form of rigorous clinical trials such as double-blind studies, is essential to validate the efficacy of glutamine in humans and further elucidate its intricate mechanisms of action.

### β-alanine

β-alanine is an ergogenic aid favored by athletes for its potential to enhance performance. This non-essential β-amino acid, distinct in being one of the naturally occurring endogenous β-amino acids in both humans and animals, is synthesized through three main pathways [175–177]. First, it can be produced through the decarboxylation of L-aspartate, a reaction catalyzed by aspartate decarboxylase, which is secreted by gastrointestinal microbes. Secondly, β-alanine can emerge as a byproduct of the reaction between L-alanine and pyruvate, facilitated by β-alanine-pyruvate transaminase. Lastly, it is also generated through the decarboxylation and deamination of uracil, which involves three enzymes: dihydropyrimidine dehydrogenase, dihydropyrimidinase, and 3-Ureidopropionase.

Although β-alanine does not directly participate in synthesizing proteins or enzymes, it is physiologically significant holds. Its importance is primarily attributed to its role in the synthesis of carnosine (β-alanyl-L-histidine). This dipeptide is formed in muscle through a reaction between β-alanine and L-histidine, catalyzed by carnosine synthase with the involvement of ATP [178]. Carnosine, synthesized in this manner, is distributed throughout various organs, including the muscles, the brain, and the central nervous system [179]. Muscle carnosine serves multiple functions, including acting as an intracellular pH buffer, chelating metal ions, inhibiting glycation, and functioning as an antioxidant [180].

### The potential mechanism of β-alanine in regulating skeletal muscle metabolism

According to recent research, the supplementation of β-alanine has been shown to enhance performance, improve muscle function and decrease fatigue during exercise [181, 182]. β-alanine is essential in exercise due to its role as a rate-limiting reactant in the synthesis of carnosine, and exogenous supplementation of β-alanine effectively elevate muscle carnosine concentrations [183–185].

The mechanisms underlying the action of carnosine encompasses several important aspects (Fig. 1). Firstly, carnosine functions as a pH buffer, mitigating the accumulation of lactate during physical activity and thereby mitigating acidosis and its negative impact on exercise performance and fatigue [186–188]. Secondly, carnosine demonstrates antioxidant properties through mechanisms that are both metal-ion-independent and metal-ion-dependent, providing protection against exercise-induced ROS damage [189–191]. Furthermore, carnosine has a high affinity for binding to the imidazole group of carnosine, facilitating its rapid oxidation and resulting in a decrease in levels of ROS in the presence of copper [15, 192]. Thirdly, carnosine functions as a protective agent against the formation of advanced lipoxidation end-products (ALEs) and advanced glycoxidation end-products (AGEs) by inhibiting protein carbonylation and glycoxidation [193–196].This protective mechanism primarily involves the prevention of lipid or glucose oxidation and the generation of reactive carbonyl species (RCS), thereby safeguarding cells from potential damage [15, 197, 198]. Lastly, carnosine serves as a modulator of calcium release and calcium sensitivity, playing a role in the regulation of muscle excitation and contraction coupling, ultimately improving muscle function [199, 200].

In summary, the primary function of β-alanine is to synthesize carnosine, which may regulate skeletal muscle metabolism by acting as intracellular pH buffer metal-ion chelator, and regulator of calcium release and calcium sensitivity (Fig. 1). These multifaceted mechanisms underscore the significance of carnosine as a beneficial component in improving exercise performance and muscle function.

### Dosage recommendations and adverse effects

Since β-alanine is the rate limiting precursor for carnosine synthesis, it is necessary to consume 1–3 g of β-alanine daily through dietary sources [49]. In the context of athletes and sportsmen, the recommended dosage range for β-alanine supplementation is 3.2–6.4 g/day (0.04–0.08 g/kg/day, for an 80 kg individual) [201–203]. Numerous studies consistently demonstrate that supplementing with 4–6.4 g of β-alanine per day for a period of 4–10 weeks can lead to a significant increase in muscle carnosine content ranging from 40–100% [187, 204]. According to Harris et al., an increase of 40% in muscle carnosine levels is associated with a 4% enhancement in overall muscle buffering capacity and a 7% improvement in buffering capacity specifically in type II muscle fibers [185].

The most recent recommendation by Sport Integrity Australia (SIA) suggests initiating β-alanine supplementation at a dosage of 3.2 g/day for 8 weeks or 6.4 g/day for 4 weeks, followed by a maintenance dose of 1.2 g/day [205]. In this sense, supplementation of 1.2 g/day β-alanine has been demonstrated to be the optimal dose for sustaining muscle carnosine levels at 30–50% above pre-supplementation levels [206]. A meta-analysis revealed that the duration of exercise significantly influences the efficacy of β-alanine supplementation. Specifically, exercise periods lasting between 0.5 and 10 min are likely to yield the greatest benefits [182]. Moreover, when compared to the supplementation of β-alanine alone, the combined supplementation of β-alanine and sodium bicarbonate has been shown to produce the most significant effect [182].

A meta-analysis assessing the comprehensive risk of toxicity concluded that β-alanine supplementation (6.4 g/day for 24 weeks) poses no adverse effects in healthy individuals [203, 207]. Additionally, recent studies have shown that supplementation with 12 g/day of β-alanine has no side effects in the short term (7 or 14 days) [208, 209]. However, the establishment of a safe upper intake level for β-alanine remains a subject requiring further investigation.

### Limitations and future research opportunities

Research has found that direct supplementation of carnosine does not produce the equivalent outcomes as β-alanine due to the swift degradation of carnosine by serum carnosinase into β-alanine and histidine upon ingestion, diminishing its efficacy [15, 210]. Consequently, the development of carnosine analogs that are resistant to degradation by carnosinase represents a significant challenge for future research. In addition, supplementing histidine in isolation did not significantly increase the level of muscle carnosine [211]. However, prolonged β-alanine supplementation may diminish the available pool of free histidine in muscles or plasma, suggesting that the co-supplementation of histidine and β-alanine may be a promising strategy for increasing carnosine levels [211].

Furthermore, research indicates that females naturally possess lower levels of intramuscular carnosine than males, with their carnosine content potentially increasing at a faster rate following β-alanine consumption [212, 213]. This finding suggests that females may exhibit different responses to β-alanine administration. However, there is a lack of research regarding the impact of β-alanine on female athletes. It remains to be determined whether females may require different β-alanine supplementation regimens in comparison to males and how β-alanine influences female body composition and muscle functions in relation to its ergogenic properties.

## Conclusions and perspectives

Amino acids are widely used dietary supplements by individuals engaged in sports training and physical activity. This review outlines the effectiveness, recommended dosage, potential adverse effects (Table 1) and mechanisms (Fig. 1) of specific amino acids, including BCAA, creatine, glutamine and β-alanine, in regulating skeletal muscle metabolism. Significantly, existing evidence indicates that supplementation with these amino acids at prescribed doses is typically safe for healthy adults. Further research is necessary to investigate the adaptive responses and potential adverse effects linked to high-dose and prolonged amino acid supplementation. Furthermore, this review discusses the existing gaps in current literature. Future studies should to elucidate the impacts and optimal dosage schedules of amino acids across various demographic groups, considering variables such as sex, age, type and duration of physical activity, as well as specific physiological conditions like pregnancy, lactation, and hepatic or renal disorders. In conclusion, this review offers valuable insights for elite athletes and fitness enthusiasts, providing recommendations on the selection and utilization of specific amino acids in dietary supplementation to promote the maintenance and improvement of skeletal muscle mass.

**Table 1:** The effects and dosage recommendations of amino acid supplementation in healthy and physically active individuals

	Effectiveness	Recommended dosage	Upper limit dosage	Adverse effects
**BCAA**	increases LBM in individuals who do not meet recommended protein intakes for exercise	0.144 g/kg/day for healthy adults	0.5 g/kg/day for adults; 0.43 g/kg/day for individual aged over 70 years	increased blood ammonia concentration if exceeding upper limit
**creatine**	increased LBM and muscle strength when combined with resistance training	5–10 g/day (about 0.07–0.14/kg/day for an 70 kg individual) for physically active adults to sustain increased creatine stores	has not been identified	0.3 to 0.8 g/kg/day for up to 5 years has no adverse health risks
**glutamine**	no relationship with LBM	2–40 g/day (about 0.05–0.6/kg/day for 70 kg individual) for physically active adults	has not been identified	Glutamine is safe and well-tolerated at recommended doses
**β-Alanine**	improves intramuscular synthesis of carnosine, intracellular buffering capacity and contractile function of skeletal muscle	3.2–6.4 g/day (about 0.04–0.08 g/kg/day) for active adults engaging in 0.5–10 min bouts of intense exercise	has not been identified	up to 12 g/day for 7 or 14 days has no adverse health risks

## Data Availability

No datasets were generated or analysed during the current study.
